# Role of orthobiologics in managing patellar tendinopathy: A narrative review

**DOI:** 10.1002/jeo2.12099

**Published:** 2024-07-24

**Authors:** Sumit Banerjee, Ragulajay Balamarthandapuram Gopalakrishna, Abhay Elhence

**Affiliations:** ^1^ Department of Orthopedics All India Institute of Medical Sciences Jodhpur Rajasthan

**Keywords:** Jumper's knee, orthobiologics, patellar tendinopathy, platelet‐rich plasma, stem cells

## Abstract

Patellar tendinopathy is a relatively common cause of anterior knee pain in athletes. It is predominantly seen in sports involving jumping, running, abrupt change in direction like basketball, volleyball, soccer, sprinting and jumping. The main pathophysiology is considered to be repetitive microtrauma at the inferior pole of patella along with a poor healing response. Although eccentric exercises have shown to be beneficial, the improvement is often slow and may result in a less than satisfactory outcome. There is a growing interest of orthobiologics, mainly platelet‐rich plasma (PRP) in multiple chronic musculoskeletal pathologies. This narrative review aimed to analyse the current evidence on the role of orthobiologics in the management of recalcitrant patellar tendinopathy. Multiple studies have shown significant clinical improvement with negligible adverse effects on PRP injection for patellar tendinopathy. Most studies assessed report that the effects of PRP are sustained. However, among all studies evaluated, there is a considerable heterogeneity in terms of PRP composition, number of injections, dosage interval and postinjection rehabilitation protocol, pointing to the need for further research to enable standardisation of PRP therapy. Stem cells too have shown potential to be effective as a treatment modality for chronic patellar tendinopathy, but there is limited data to recommend its use outside of research setting or to enable a meaningful comparison to PRP. There is a promising role of orthobiologics in management of chronic patellar tendinopathy not responding to conventional treatment.

**Level of Evidence:**

Not applicable (narrative review).

**Abbreviations**
ACP: autologous conditioned plasma
IGF: insulin‐like growth factor
MSC: mesenchymal stem cell
PDGF: platelet‐derived growth factor
PICO: population intervention comparison outcome
PRP: platelet‐rich plasma
RCT: randomised controlled trial
TGF‐1: transforming growth factor‐beta 1
TGF‐2: transforming growth factor beta 2
USG: ultrasonography
VAS: visual analogue scale
VISA‐P: Victorian institute of Sport assessment‐pain

## INTRODUCTION

Patellar tendinopathy is an affliction causing anterior knee pain. It has been found to be associated with sports involving jumping, such as basketball and athletics [30]. Literature suggests that chronic repetitive motion is the most significant risk factor [22], and an interplay with intrinsic risk factors is implicated in its causation. The pathological hallmark entails progressive degeneration of the tendon, paucity of tissue repair and an absence of inflammatory cells [4, 17]. Although physical therapy mainly eccentric exercises has shown to be beneficial, the improvement is often slow and incomplete and may result in less than satisfactory outcomes. Chronic recalcitrant patellar tendinopathy often requires surgery, which carries its own risks and a delayed return to sports activities [5].

The advent and growing popularity of biologicals can be seen in their use as a treatment option in managing patellar tendinopathies. Platelet‐rich plasma (PRP) is autologous plasma that has been centrifuged to concentrate platelets at least two to three times from native concentration [28]. The high level of growth factors, such as transforming growth factor‐beta 1 (TGF‐β1), transforming growth factor‐beta 2 (TGF‐β2), insulin‐like growth factor (IGF), platelet‐derived growth factor (PDGF), and so on, have been postulated to account for its therapeutic importance [14]. The role of PRP in patellar tendinopathies has been investigated by multiple authors, and the reported results have been mixed [3, 15, 25]. A more recent inclusion in the management arsenal has been the use of mesenchymal stem cells (MSC). Stem cells are autologous cells that have the potential to differentiate/transform into cells from multiple lineages. They have been hypothesised to help tendon remodelling by accelerating healing and improving the quality of the regenerated tendon. The literature pertaining to the use of stem cells in patellar tendinopathy is scant and is mainly confined to in‐vitro studies [8], animal studies [23] or case series [27]. While biologicals have proven effective in patellar tendinopathies, high‐level evidence comparing their efficacy is yet to be readily available. The purpose of this narrative review is to summarise the current evidence in literature on the role of orthobiologics as an option for the management of patellar tendinopathy.

### PRP in patellar tendinopathy

PRP is an important part of the armamentarium of orthobiologics and regenerative orthopaedics. It is increasingly used in the biological management of various musculoskeletal injuries of ligaments, tendons, cartilage and bone. The platelets contain alpha granules that are rich in several growth factors, such as PDGF, TGF‐β, IGF, vascular endothelial growth factor and epidermal growth factor which play key roles in tissue repair mechanisms [9]. The aetiology of patellar tendinopathy is considered due to a failed or inadequate healing response to microtrauma [3]. The rationale of PRP injection use is that the high concentration of growth factors will enhance the tissue healing response. PRP injections have emerged as one of the most important nonoperative modalities in the management of chronic patellar tendinopathy. The current literature with its study characteristics and outcome of PRP in patellar tendinopathy is tabulated in Table 1.

**Table 1:** Study characteristics and outcome of current literature on PRP in patellar tendinopathy.

References	Type of study	Methodology	Sample size and mean age	Follow‐up period (minimum/mean/standard)	Clinical outcome scores (baseline, final follow‐up postinjection)	Return to sports/presymptom activity level
Kon et al. [19]	Prospective, noncomparative	3 injections of PRP injected intra and peri‐tendinous	20, Mean age 25.5 years	6 months (minimum)	Tegner, EQ‐VAS SF‐36 (absolute values—NR) Statistically significant improvement in all three scores	NR
Charaousset et al. [6]	Prospective, noncomparative	3 injections of PRP injected into patellar tendon lesion.	28, Mean age 27 years	24 months (minimum)	VISA‐P (39–94) VAS (7–0.8) Lysholm score (60–96) Statistically significant improvement in all scores	75% Returned to sports at mean of 12 weeks.
Ferrero et al. [11]	Prospective, noncomparative	2 intratendinous PRP injections into lesions	24 patients (28 tendons) Mean age—NR	6 months (standard)	VISA‐P 57 (baseline) to 75.5 (6 months) Statistically significant improvement	71% (good/excellent satisfaction) Average return to sports at 6 weeks
Zayni et al. [36]	Prospective, comparative study	Single PRP injection vs. two PRP injection in chronic patellar tendinopathy	40 (20 in each cohort) Mean age 24.4 years	34 months (mean) 24 months (minimum)	Single injection group VISA‐P (36.7–65.7) VAS (7.1–3.6) Tegner score (4.1–5.9) Two injection group VISA‐P (35.7–93.2) VAS (6.7–1.0) Tegner score (4.8–8.1) Statistically significant improvement in all scores in both groups, more in two injection group	86% (presymptom activity level) Average time to return to sports 6.7 weeks
Filardo et al. [12]	Prospective, noncomparative	Three intratendinous PRP injection in chronic patellar tendinopathy	43 (Mean age 30.6 years)	48.6 months (mean) (minimum 36 months)	VISA‐P (44.1 at baseline to 84.3 at 4 years) EQ‐VAS (67.8 at baseline to 85.2 at 4 years) Tegner (3.3 at baseline to 5.7 at 4 years) Statistically significant improvement in all scores	81.4% (return to sports)
Kaux et al. [15]	Prospective, noncomparative	Single intratendinous PRP injection	20 Mean age 28.8 years	12 months (standard)	VISA‐P (47.9 at baseline to 74.2 at 1 year) IKDC (52.7 at baseline to 77.0 at 1 year) VAS (6.3 at baseline to 1.4 at 1 year) Statistically significant improvement in all scores at 1 year	70% (return to sports)
Vetrano et al. [34]	Prospective, comparative	Comparative study between Two intratendinous PRP injection and ESWT (3 sessions)	46 (23 patients in each cohort)	12 months	PRP group VISA‐P (55.3 at baseline to 91.3 at 1 year) VAS (6.6 at baseline to 1.5 at 1 year) PRP group showed statistically significant improvement at year compared to baseline in VAS, VISA‐P and modified Blazina grading. The difference in improvement in PRP group compared to ESWT group was statistically significant	91% (return to preinjury sports)
Gosens et al. [13]	Prospective, comparative	Comparison of efficacy of intratendinous PRP injection in patellar tendinopathy in Patients who had received prior injection or surgery (group 1) vs those who had not received any prior treatment (group 2)	36, Group 1 (received prior injection/surgery)—14 Group—2 (no prior treatment)—22 Mean age (overall)—30.9 years	18 months	Overall result (both groups combined) VISA‐P (40.1–57.7) VAS ADL (5.9–2.7) VAS work (6.3–3.2) VAS sport (8.5–4.6) Assessed overall there was a statistically significant improved in all scores. When assessed group wise, Group 1 (received prior treatment) VISA‐P improvement was not statistically significant, although all VAS scores improvement was statistically significant. The inference is intratendinous PRP injection has clinically significant efficacy in chronic patellar tendinopathy, although the improvement may be comparatively less if treated prior with other injections or surgery.	NR
Abdelbary and Bassiouny [2]	Prospective, comparative	Comparison of efficacy of Single intratendinous PRP injection vs. high volume image guided injection (saline 30 mL, 0.5% lignocaine 10 mL, 25 mg hydrocortisone)	20 (10 patients in each cohort) Mean age—NR Range: 28–45 years	12 months	VAS was assessed at end of 1 year. 7/10 patients treated with PRP had no pain and 3/10 patients had mild pain. Statistical analysis—not done due to small sample size.	NR
Abate et al. [1]	Prospective, comparative	Comparative study between 3 groups PRP, HVIGI PRP + HVIGI	54 patients (18 in each cohort)	3 and 6 months (standard)	VAS and VISA‐P evaluated at 3 and 6 months. At 3 months, Both PRP and HVIGI showed similar improvement in scores. However, at 6 months the improvement in HVIGI group reduced gradually. PRP + HVIGI showed highest improvement in VAS and VISA‐at 3 and 6 months.	NR

Abbreviations: ADL, activities of daily living; EQ‐VAS, EuroQol‐ Visual Analogue Scale; ESWT, extracorporeal shock wave therapy; HVIGI, high volume image guided injections; IKDC, International Knee Documentation Committee; PRP, platelet‐rich plasma; SF‐36, Short form Questionnaire; VISA‐P, Victorian Institute of Sport assessment‐pain; VAS, visual analogue scale.

Scott et al. [31] did a randomised controlled trial (RCT) on 57 patients with patellar tendinopathy. They were divided into three cohorts (19 patients in each) and were managed with leucocyte‐rich PRP (LR‐PRP), leucocyte‐poor PRP (LP‐PRP) and saline. At the end of 12 weeks, 58% of all patients had improvement in their Victorian Institute of Sport Assessment–Patella (VISA‐P) scores regardless of the assigned group. There was no statistically significant difference between PRP (LR or LP) and saline.

Manfreda et al. [25] studied the efficacy of ultrasonography (USG) guided PRP injection in 17 athletes with patellar tendinopathy. At 4 and 12 months follow‐up, there was poor improvement in VAS score, no improvement in Tegner score and statistically insignificant improvement in VISA‐P score. They concluded that there are doubts in the real use of PRP in tendinopathies and requires further investigation.

Multiple studies [1, 2, 6, 11, 12, 13, 15, 19, 31, 34, 36] have proven efficacy of PRP in the management of chronic patellar tendinopathy refractory to nonoperative management as mentioned in Table 1. However, none of the implemented PRP formulations for patellar tendinopathy can be replicated due to the lack of information on its composition in the presented studies.

The return to sports or presymptom activity level was reported from 70% [9] to 91% [21] among various studies. Majority of the studies had used multiple (>2) doses of USG‐guided intratendinous PRP, while study by Zayni et al. [36] compared the efficacy of one versus two injections. The time interval between injection varied among studies from a minimum of 5 days to a maximum of 3 weeks while bi‐weekly was the most common interval followed. The PRP preparation protocol had significant variability among studies in terms of volume of venous blood taken, anticoagulant used, centrifugation technique and the centrifugation system used. The detailed methodology of PRP preparation and administration technique in each evaluated study is shown in Table 2.

**Table 2:** Detailed methodology and technique of PRP administration and rehabilitation protocol.

References	Type of PRP	System used and methodology	Volume and platelet concentration	Activator used	Number of injections, interval between injections	USG guidance	Rehabilitation protocol
Kon et al. [19]	Pure	150 mL venous blood with 21 mL sodium citrate, centrifuged twice (first 1800 rpm for 15 min, second 3500 rpm for 10 min 20 mL PRP (split into 4 units of 5 mL each, 1 unit for lab anlsis, rest 3 units for injection in 3 settings)	5 mL (each injection) 6.8 million platelets (per injection)	10% calcium chloride	3, every 15 days	No	Rest after first injection Stretching and mild activities after second and third injection Sports or recreational activities as tolerated after 1 month.
Charaousset et al. [6]	Pure (leucocyte poor)	Arthrex ACP system 15 ml venous blood, Centrifuged at 1700 rpm for 5 minutes	6 mL Concentration –NR	None used	3, weekly	Yes	Warm up and stretching exercises, Followed by Eccentric exercise programme described by Stanish et al. [33], Running from 6^th^ week after last injection and sports from eighth week.
Ferrero et al. [11]	NR	NR	6 mL, concentration—NR	NR	2, 3 + 0.52 weeks	Yes	No physical activities till 2 weeks after injection Physiokinesiotherapy after 2 weeks with gradual return to sports
Zayni et al. [36]	Pure	Arthrex ACP system 15 mL venous blood centrifuge at 1700 rpm for 5 min	6 mL, concentration—NR	None used	Comparative study of single vs. two injections (2 weeks interval)	Yes	Rest for 2 weeks, followed by rehabilitation programme which included cycling, electric stimulation and eccentric exercise as described by Stanish et al. [33], and Kaux et al. [16]
Filardo et al. [12]	NR	150 mL venous blood centrifuged twice (1480 rpm at 6 min, followed by 3400 rpm for 15 min) 20 mL PRP split into 4 units (5 mL each)—1 unit for lab analysis, 3 units for injection	5 mL, concentration—NR	10% calcium chloride	Three, 2 weekly	Yes	Rehabilitation programme started after second injection —eccentric exercises for 12 weeks Sports encouraged after 12 weeks.
Kaux et al. [15]	Pure	Comtec Frensius kabi apheresis machine	6 mL, 8–9 × 10^5^/μL	Calcium chloride	Single	No	1‐week rest postinjection followed by standardised submaximal eccentric exercises Sports gradually started 6 weeks postinjection
Vetrano et al. [34]	NR	Kaylight LTD 10 mL blood with acid‐citrate‐dextrose Centrifuged at 1500 rpm for 15 min, 1 mL PRP sent for lab analysis	3–5 mL 0.89–1.1 × 10^9^/mL	None used	Two, weekly	Yes	1‐week rest postinjection followed by standardised stretching and muscle strengthening protocol for 2 weeks. Gradual return to previous training after 4 weeks Sports return individualised as per pain tolerance and clinical signs of patient.
Gosen et al. [13]	Leucocyte‐rich PRP + 0.5% Bupivacaine + epinephrine	Recover system (Biomet Biologics) 27 mL blood mixed with 3 ml sodium citrate (other details regarding centrifugation not reported) PRP buffered to physiological pH using sodium bicarbonate, 1 mL 0.5% bupivacaine and 1 mL epinephrine (1:200,000) added	3 mL Concentration—NR	None used	Single	No	24 h postinjection Standardised stretching exercises for 2 weeks started, Followed by eccentric exercises. Return to sports or recreating activities after 4 weeks as tolerated.
Abdelbaryand Bassiouny (comparative study of PRP vs. HVI) [2]	NR	Arthrex system, 15 mL venous blood centrifuged at 1700 rpm for 6 min.	6 mL, concentration—NR	None used	Single	Yes	4 days rest postinjection, Loading started from fifth day, Cardiovascular activities from eighth day Sports started after 13th day depending on pre‐injection status.
Scott et al. [31]	LR and LP PRP	Angel Cytomedix system LP‐PRP and LR‐PRP prepared separately by setting haematocrit to 2% and 15%, respectively 52 mL venous blood mixed with 8 mL citrate—dextrose anticoagulant.	3.5 mL, 3.8 times of normal count for LR‐PRP, 3.0 times for LP‐PRP (absolute values NR)	None used	Single	Yes	1‐week postinjection, Slow heavy rehabilitation programme comprosed of concentric and eccentric exercises as described by Kongsgaard et al. [20] started
Manfreda et al. [25)	Pure	GPS II 50 mL blood mixed with calcium gluconate (1:10 of calcium gluconate to blood ratio) and centrifuge to get PRP Sodium bicarbonate added to PRP to bring it to physiological pH.	5 mL, concentration—NR	None used	Three, 15 days	Yes	Rest for 1 week, Walking in water and swimming at moderate speed from 7 to 21 days, Eccentric (quadriceps) and concentric (leg flexors) exercises from 5th week. Sports‐specific exercises started at 9th week and Return to Sports from 12th week
Dragoo et al [10]	Leucocyte‐rich	GPS3 (Biomet) 55 mL of venous blood processed (details of centrifugation method—NR) Subcutaneous injection of 3 mL 0.25% bupivacaine with 1:100,000 epinephrine injected subcutaneously, followed by dry needling (10 times) of tendinopathy area, then PRP was injected intratendinous	6 mL, concentration—NR	NR	Single	Yes	Standardised five‐phase eccentric exercise programme (details—NR)

Abbreviations: ACP, autologous conditioned plasma; GPS, gravitational platelet concentration system; HVI, human immunodeficiency virus; LP‐PRP, leucocyte‐poor PRP; PRP, platelet‐rich plasma; USG, ultrasonography.

Most of the studies had not used any activator agents while three studies [12, 15, 19] used 10% calcium chloride as a PRP activator agent. Scott et al. [31], had prepared LR PRP and LP PRP separately and analysed the efficacy of both, while Charaousset et al. [6] had used LP‐PRP and Dragoo et al. [10], LR‐PRP. Other studies did not report the leucocyte content of PRP. The most commonly used outcome measures in patellar tendinopathy studies were the VISA‐P, Visual Analogue Scale (VAS) and Tegner scale. The other scales used were Modified Blazina, EuroQol‐ Visual Analogue Scale (EQ‐VAS), Short form Questionnaire, International Knee Documentation Committee (IKDC) and Lysholm score.

### How long does PRP injection work?

One study by Dragoo et al. [10] reported dissipation of the beneficial effects of PRP after 26 weeks. However, multiple other studies [6, 12, 15, 19, 34, 36] had reported a constant improvement in outcome scores till final follow‐up. Study by Filardo et al. [12] had the maximum reported mean follow‐up of 48 months with a minimum follow‐up of 36 months. They had reported constant improvement in the outcome score (VISA‐P) at every follow‐up up to 4 years. Hence, beneficial effects of PRP injection can be considered to have reasonable mid‐term duration of action.

### Imaging follow‐up post‐PRP injection

Charaousset et al. [6] was the only study which had reported a magnetic resonance imaging (MRI) evaluation on the follow‐up post‐PRP injection. They had reported complete return of structural integrity of patellar tendon in 57% cases and partial healing in 43% of cases. This imaging finding also had clinical correlation, in that all of the six patients considered to be treatment failures had only partial healing on MRI. Three studies [1, 11, 12] had reported USG/Doppler evaluation in follow‐up period. Ferrero et al. [11], found decrease in tendon thickness and hypoechoic areas and improvement in fibrillar echotexture suggestive of tendon regeneration with a decrease in hypervascularity in the tendon. Filardo et al. [12] in his study found increase in the patellar tendon thickness and vascularity after first two injections followed by gradual decrease of both. The vascularity reached the baseline (preinjection) level at 6 months and was lower than baseline at the final follow‐up. Similar finding was reported in a study by Abate et al. [1], who found a decrease in neo‐vascularisation of the patellar tendon at 6 months post‐PRP injection. However, no significant change in tendon echo‐texture was found in their study.

### Adverse effects/complications of PRP injection

None of the studies had reported any serious adverse effect or complication following intratendinous PRP injection. Most of the reported adverse effects were mild pain and discomfort which subsided shortly with oral analgesics and ice packs. Kon et al. [19], reported one patient with marked pain response that lasted for 3 weeks. Hence, it can be inferred from multiple studies that PRP being an autologous product is safe in majority of the patient population. However, it is worthy to note that many authors had excluded the following population from their study [1, 6, 11, 12, 19, 34]—diabetes, rheumatoid arthritis, coagulopathies, cardiovascular disease, infection, immunosuppression, patients on anticoagulants/antiaggregants, haemoglobin <11 g%, platelet count <1,50,000/mm^3^ and pregnancy. Hence, safety is these patients is not proven.

### Multiple factors affect the efficacy of PRP injection in patellar tendinopathy

#### Evidence of current literature on these factors

(1) Platelet concentration
  Few studies have mentioned the platelet concentration used, although in different units. Kon et al. [19] had mentioned 6.8 million/injection, Kaux et al. [15] 0.8–0.9 × 10^6^/μL, Vetrano et al. [34], 0.89–1.1 × 10^6^/μL and Scott et al. [31], had mentioned as 3.8 times the normal concentration for LR‐PRP and 3.0 times the normal concentration for LP‐PRP, although the absolute values were not mentioned. None of the human studies on PRP in patellar tendinopathy had studied the effect of platelet concentration in PRP on the outcome. One animal study on induced patellar tendinopathy in rats by Yoshida et al. [35] had reported that platelet concentration of 1.0 × 10^6^ was more effective in pain relief and clinical improvement compared to a concentration of 5 × 10^5^, while there was no difference in histopathology.
(2) Leucocyte concentration
  LR‐PRP versus LP‐PRP leucocyte concentration is one of the key characteristics in differentiating PRP formulations. There are concerns expressed in studies that the pro‐inflammatory effect of leucocytes in PRP may affect its function. On the other hand, some studies encourage the use of LR‐PRP as the inflammatory process is also a part of healing known as ‘inflammatory regeneration’ and platelet–leucocyte interaction may be necessary for optimal tissue healing [21].
  Zhou et al. [37] studied the effect of LR‐PRP versus LP‐PRP in tendon stem cells (TSCs) isolated from the patellar tendon of rabbits. The expression of Stem cell markers, inflammatory genes, catabolic and anabolic proteins were studied. They found that both LR PRP and LP‐PRP induced the proliferation and differentiation of TSCs into active tenocytes.
  However, they found that LR‐PRP may be detrimental to the healing of injured tendons due to the catabolic and inflammatory effects on tendon cells. On the other hand, LP‐PRP may induce excess scarring in acutely injured tendons due to its anabolic effect. They suggested that LR‐PRP versus LP‐PRP should be decided based on the duration of the injury. They suggested that LR‐PRP may be utilised in acute conditions and LP‐PRP in chronic conditions. Another animal study was conducted by McCarrel et al. [26], comparing the effect of PRP with three varying leucocyte concentrations (intermediate leucocyte concentration [standard] PRP, LR‐PRP and LP‐PRP). Explants of Flexor digitorum superficialis tendons of young adult horses were cultured in the three PRP groups with varying leucocyte concentrations and expression of inflammatory markers were studied. They found higher expression of inflammatory cytokines in LR PRP group. This laboratory animal study finding was extrapolated to ‘leucocyte reduced (poor) PRP may be the optimum preparation to stimulate healing without scar tissue formation’. Further large‐scale randomised studies are needed to comment on the effect of leucocyte concentration on PRP efficacy.
(3) Number of injections
  Majority of the studies [1, 6, 8, 12, 19, 25, 34] had used multiple (≥2) PRP injections, few used single injection [2, 13, 15]. Study by Zayni et al. [36] compared the efficacy of one versus two injections in patellar tendinopathy and found two‐injection cohort to have statistically significant better outcome scores (VISA‐P, VAS, Tegner scale) compared to single injection group.
(4) Time interval between injections
  The time interval between injection varied among studies from a minimum of 5 days [25] to a maximum of 3 weeks [11]. Time interval was 2 weekly in three studies [12, 19, 36] and weekly in two studies [6, 34].

### Stem cells in patellar tendinopathy

Stem cells are undifferentiated biological cells capable of proliferation, self‐renewal, conversion to differentiated cells and regenerating tissues [24]. They are multipotent and located among specialised tissues with a primary function of their maintenance and repair.

MSCs are a type of adult stem cells and have the ability to develop into any mesodermal tissue. Thus, they can be prompted to form precursor cells to develop into tissues including bone, cartilage, muscle, tendon and ligament.

Animal studies [23] have proved efficacy of stem cells in tendon injury models. Pascual‐Garrido et al. did a clinical study [27] on the efficacy of bone marrow mononuclear stem cells (BM‐MNC) in chronic patellar tendinopathy. Eight patients with chronic patellar tendinopathy >6 months duration, confirmed with MRI were given a single intratendinous injection of BM‐MNCs harvested from anterior iliac crest. After a mean follow‐up period of 5 years, there was a statistically significant improvement in Tegner score, IKDC score and Knee injury and Osteoarthritis Outcome Score symptoms, activities of daily living and sport. Soler et al. [32], did a prospective comparative study between bone marrow‐derived mesenchymal stem cells (BM‐MSCs) and LP‐PRP in 20 patients with chronic patellar tendinopathy. In the first phase of the study, BM‐MSC injection and PRP injection were given to 10 patients each. At the end of 6 months, the BM‐MSC group showed better tendon structure and tendon gap regeneration while the PRP group did not show any significant regeneration. Subsequently, the 10 patients in PRP group were given BM‐MSC injection. All the patients were evaluated again at 12 months. The 10 patients who were originally treated with BM‐MSC continued to show statistically significant improvement in both tendon structure and clinical scores (VISA‐P and VAS). The second group who were initially treated with PRP injection and later BM‐MSC injection showed tendon regeneration on MRI and also clinical improvement. No adverse effects were reported. Khoury et al. [18], did a study on efficacy of adipose‐derived mesenchymal stromal cells (ASCs) in 14 patients with 16 knees of chronic recalcitrant patellar tendinopathy. Patients with exercise‐related pain at patellar tendon insertion >6 months duration, confirmed with MRI by patellar tendon tear or thickening and not responding to exercise‐based rehabilitation and other treatment modalities >6 months were injected with three bi‐weekly injections of autologous ASCs harvested from peri‐umbilical fat. The outcome was assessed at third, sixth, and 12th months with VISA‐P, VAS and Tegner score. There was a statistically significant improvement in each follow‐up in all the scores. MRI evaluation at 6 months postinjection showed a statistically significant improvement in tendon thickness, tear length, tear width, and tear thickness.

Clarke et al. [7], did a RCT comparing skin (dermal fibroblast) derived tenocyte‐like cells with autologous plasma in management of refractory patellar tendinosis. Tenocyte‐like cells cultured from skin biopsy and suspended in autologous plasma were injected intratendinous into areas of hypoechogenecity, fibrillation and tear. The control group received an injection of autologous plasma alone. The cell group showed a significantly greater improvement in VISA score at 6 months compared to plasma group.

There was no difference between the groups in improvement of ultrasound appearance of tendon.

### Comparison of PRP versus stem cells in patellar tendinopathy management

Literature is scarce on comparative studies between stem cells and PRP in chronic patellar tendinopathy. Only one RCT by Rodas et al. [29], was found comparing stem cells and PRP in the management of chronic patellar tendinopathy. The double‐blinded RCT was done on 20 patients with chronic proximal patellar tendinopathy (lesion > 3 mm) that failed nonoperative 224 treatment. Ten patients received intra and peri‐tendinous BM‐MSC, while others 10 received intra and peri‐tendinous LP‐PRP. The outcome was assessed clinically by VISA‐P and VAS scores and radiologically by USG and MRI at 6 months. Both groups showed statistically significant improvement in VAS and VISA‐P scores at 6 months. However, there was no statistically significant difference between the groups. BM‐MSC group showed statistically significant greater improvement in the tendon structure and ultrasound tissue characterisation in USG. Similar greater improvement in tendon structure was found in MRI in BM‐MSC group compared to LP‐PRP group.

Assumably, due to the ethical issues involved, many of the studies did not have a control group to receive a placebo. This is one of the major limitations since any improvement in the clinical condition over time cannot be completely attributed to the effect of the treatment, since improvement with time may be expected in many of the self‐limiting disorders including patellar tendinopathy. Another limitation was a limited sample size as patellar tendinopathy is not a very common pathology and usually found in athletes only.

## CONCLUSION

In multiple studies, PRP and MSCs have proven to provide significant clinical and radiological improvement in chronic patellar tendinopathy. Significant variation was found among studies in the PRP preparation and administration protocol, including preparation technique, dose, platelet concentration, leucocyte rich or depleted, anticoagulant used, activating agent used or not, single or multiple injections, USG‐guided or nonguided, the time interval between injections, postinjection rehabilitation protocol, among others. No clinically significant adverse effect was reported in any of the studies. Further large‐scale randomised studies with a standardised protocol and a control group are needed to quantify the exact efficacy of these orthobiologics in managing chronic patellar tendinopathy.

## AUTHOR CONTRIBUTIONS

Sumit Banerjee conceptualised the study, formulated and conducted the electronic search, reviewed the studies and was involved in data analysis and final manuscript preparation and editing. Ragulajay Balamarthandapuram Gopalakrishna participated in the formulation and conduction of electronic search, review of studies, data analysis and editing of the manuscript. Abhay Elhence was involved in the conceptualisation of the idea, Data analysis, Final manuscript preparation and editing.

## CONFLICT OF INTEREST STATEMENT

The authors declare no conflict of interest.

## ETHICS STATEMENT

Being a narrative review of published literature, no ethical approval was required.

## Data Availability

The data that support the findings of this study are available on request from the corresponding author.
